# Ailanthone inhibition data on seed germination and seedling growth of *Lepidium sativum* L. and *Raphanus sativus* L.

**DOI:** 10.1016/j.dib.2019.104550

**Published:** 2019-09-23

**Authors:** Sonia Demasi, Matteo Caser, Silvia Fogliatto, Francesco Vidotto, Francesco Trotta, Valentina Scariot

**Affiliations:** a Department of Agricultural, Forest and Food Sciences, University of Torino, Largo Paolo Braccini 2, 10095, Grugliasco, TO, Italy; b Department of Chemistry, University of Torino, Via Pietro Giuria, 7, 10125, Torino, Italy

**Keywords:** *Ailanthus altissima*, Allelopathy, Quassinoid, Phytotoxicity, Natural herbicide

## Abstract

Ailanthone is a quassinoid from *Ailanthus altissima* (Mill.) Swingle with allelopathic properties that deserves interest for its potential use as a natural herbicide. Data about seed germination and root and hypocotyl length of two model species (*Lepidium sativus* L.ˈIngleseˈ and *Raphanus sativus* L.ˈTondo Rosso BIOˈ) treated with different concentrations of ailanthone are reported. Data derive from experiments performed in a growth chamber on filter paper, non-sterile urban soil, and a cultivation substrate for horticulture. Part of their elaboration and interpretation can be found in the research article titled “Ailanthone from *Ailanthus altissima* (Mill.) Swingle as potential natural herbicide” (Demasi et al., 2019).

**Table:** Specifications Table

Subject area	*Plant science*
More specific subject area	*Horticulture, Crop protection*
Type of data	*Graphs and tables*
How data was acquired	*Counting of germinated seeds and measurement of root and hypocotyl length were analyzed by R software (R CoreTeam, 2017); SPSS software (SPSS Inc., version 25, Chicago, Illinois)*
Data format	*Raw and analyzed*
Experimental features	*Germination, root and hypocotyl length were recorded after treating seeds of garden cress and radish with different concentrations of ailanthone, in Petri dishes or plastic flasks, with filter paper, non-sterile soil or cultivation substrate*
Data source location	*Italy, Grugliasco (TO), facilities of Department of Agricultural, Forest and Food Sciences – University of Torino (45°03′58.5″N; 7°35′29.1″E)*
Data accessibility	*Data are available from the corresponding author upon request*
Related research article	*S. Demasi, M. Caser, F. Vanara, S. Fogliatto, F. Vidotto, M. Negre, F. Trotta, V. Scariot, Ailanthone from Ailanthus altissima (Mill.) Swingle as potential natural herbicide, Sci. Hortic., 257, 2019, 108702*[1]

**Table:** 

**Value of the Data** • Data on ailanthone herbicidal activity can serve as a benchmark for additional insights on ailanthone mode of action and bioactivity and comparisons with other allelopathic compounds. • Data on germination and growth inhibition assessed on both garden cress and radish can provide information to assess effective doses and selectivity of ailanthone for further experiments on weed management. • The dataset on ailanthone herbicidal activity can be used to compare its efficacy to that of other herbicidal compounds, both natural and synthetic.

## 1 Data

**Fig. 1:**
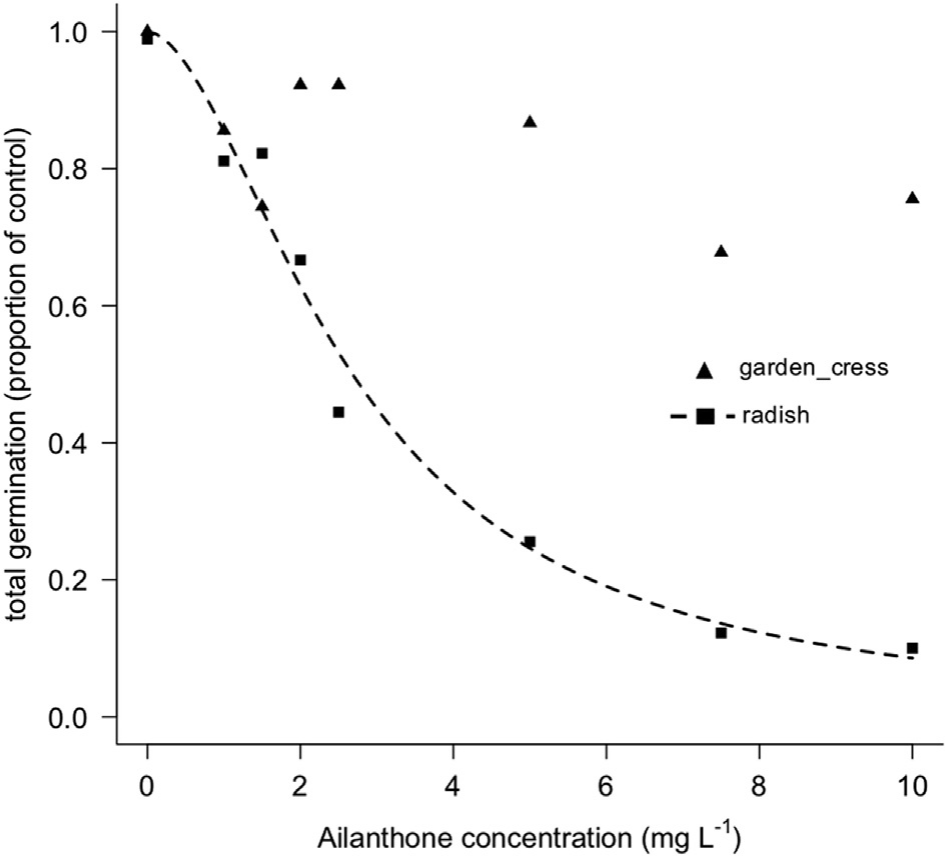
Dose-response curve of total germination (proportion of germinated seeds in the control) of radish (*Raphanus sativus*) in response to different concentrations of ailanthone in filter paper after 96 hours under controlled conditions. For garden cress, only the average values of total germination are reported, as it was not possible to draw a regression curve.

**Fig. 2:**
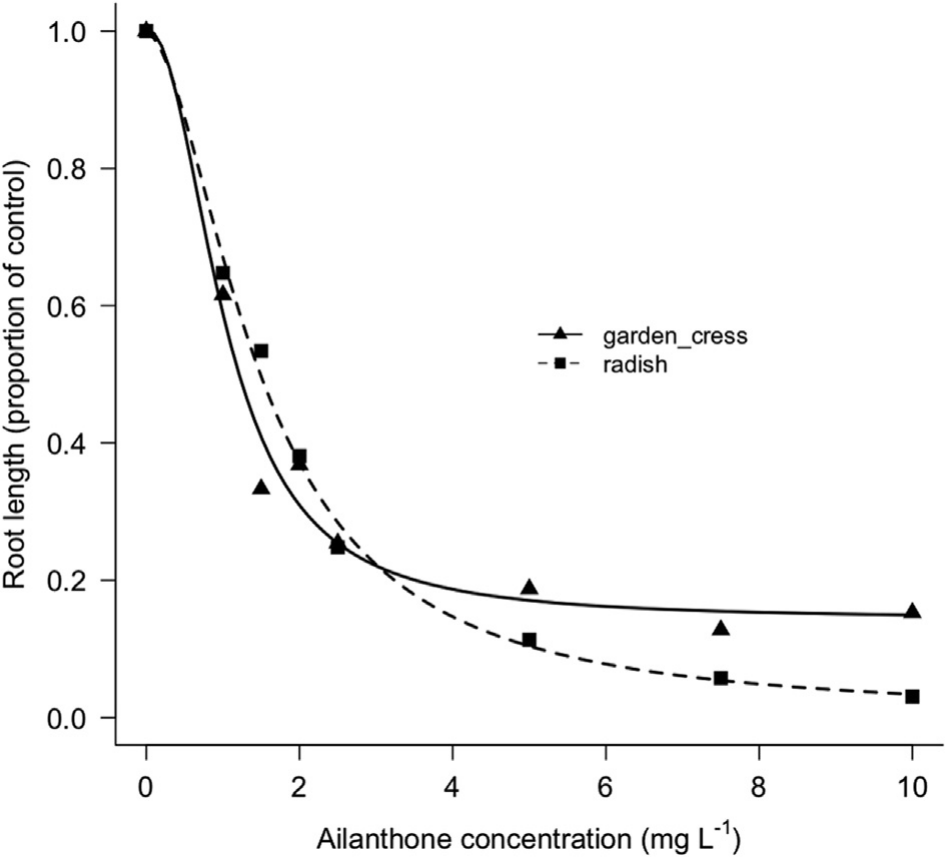
Dose-response curve of root length (proportion of control) of garden cress (*Lepidium sativum*) and radish (*Raphanus sativus*) in response to different concentrations of ailanthone in filter paper after 96 hours under controlled conditions.

**Fig. 3:**
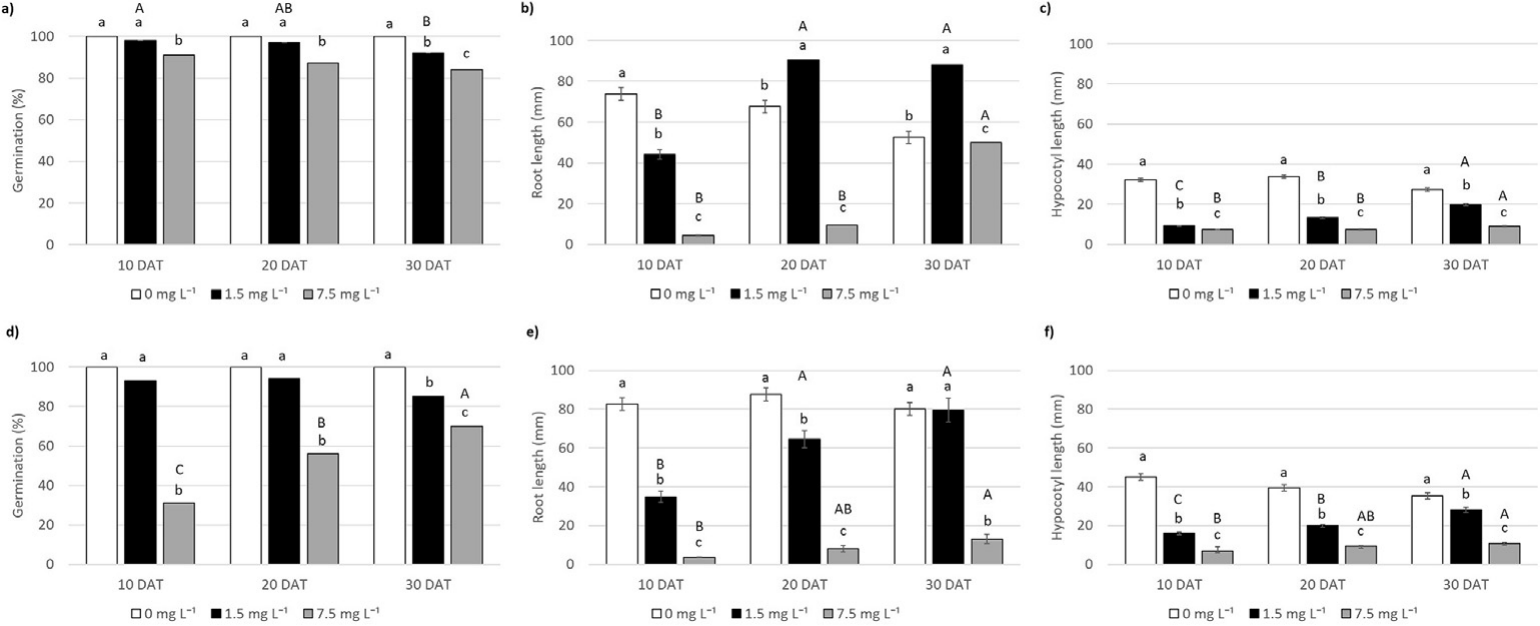
Germination (%), root length (mm) and hypocotyl length (mm) of garden cress (*Lepidium sativum*) (a, b, c) and radish (*Raphanus sativus*) (d, e, f) recorded in response to 0 mg L^−1^ (white bars), 1.5 mg L^−1^ (black bars) and 7.5 mg L^−1^ (grey bars) of ailanthone in filter paper at 10, 20, and 30 days after treatment (DAT). Different upper case letters along the same treatment or different lower case letters within the same evaluation day indicate significant differences (Tukey post-hoc test; *p* < 0.05).

**Fig. 4:**
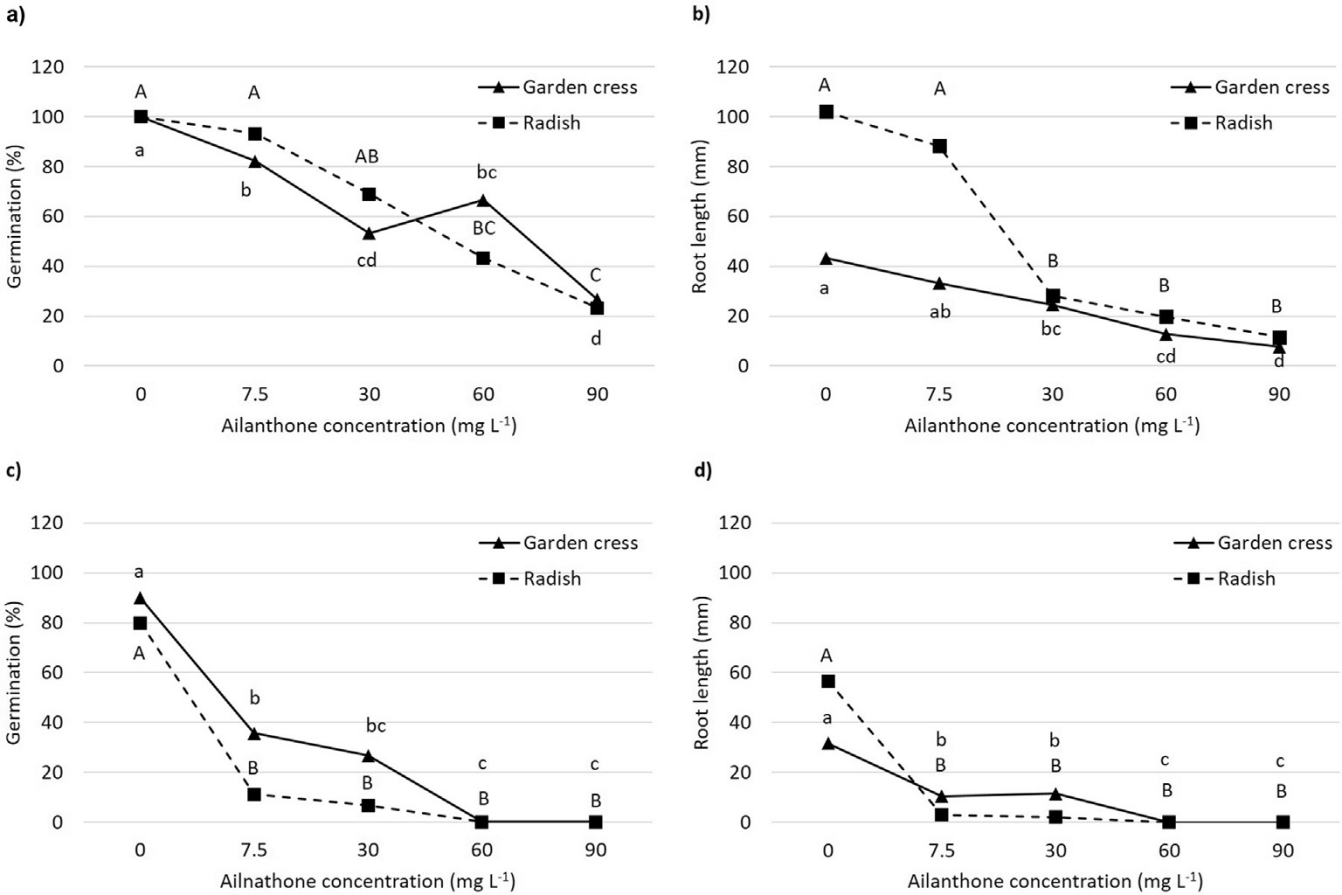
Germination (%) and root length (mm) of garden cress (*Lepidium sativum*, solid line) and radish (*Raphanus sativus*, dashed line) recorded in response to 0, 7.5, 30, 60, and 90 mg L^−1^ of ailanthone in cultivation substrate (a, b) and non-sterile soil (c, d) after 96 h. Different upper case letters in radish or different lower case letters in garden cress indicate significant differences (Tukey post-hoc test; *p* < 0.05).

**Fig. 5:**
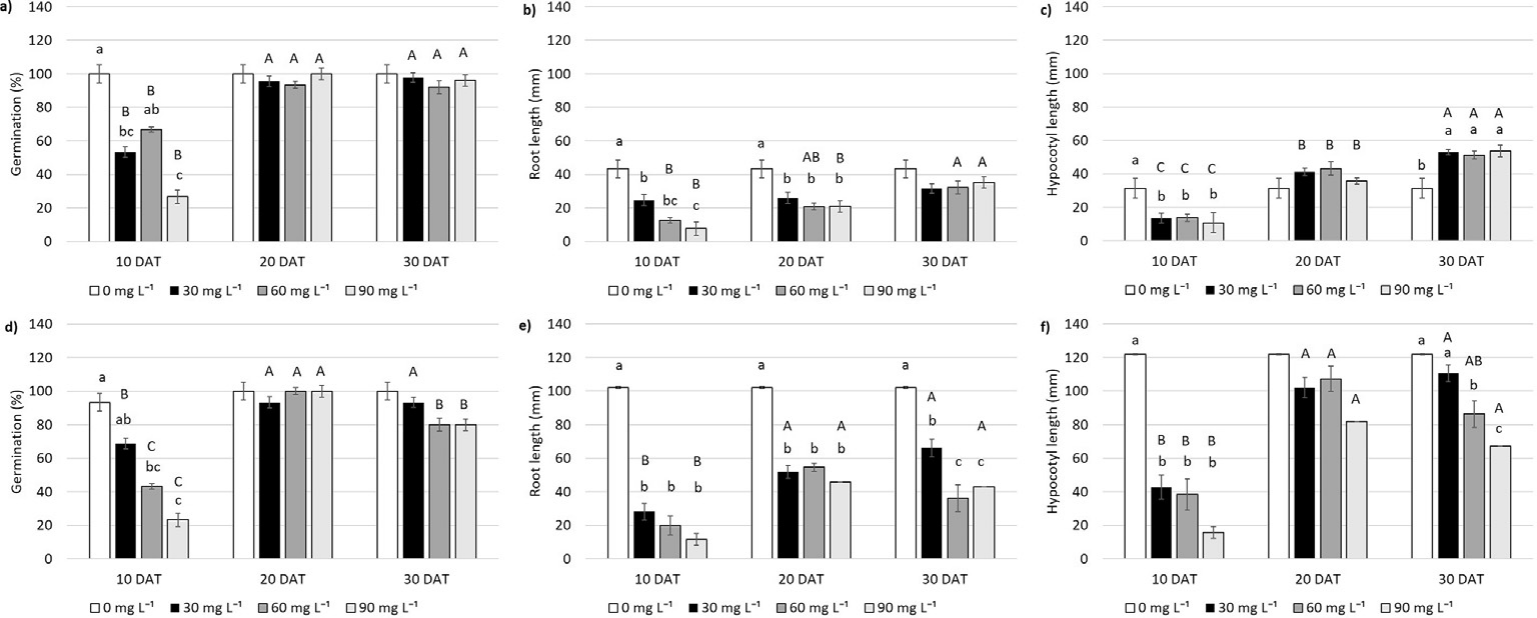
Germination (%), root length (mm) and hypocotyl length (mm) of garden cress (*Lepidium sativum*) (a, b, c) and radish (*Raphanus sativus*) (d, e, f) in response to 0 mg L^−1^ (white bars), 30 (black bars), 60 (grey bars) and 90 mg L^−1^ (light grey bars) of ailanthone in cultivation substrate 10, 20 and 30 days after treatment (DAT). Different upper case letters along the same treatment or different lower case letters within the same evaluation day indicate significant differences (Tukey post-hoc test; *p* < 0.05).

Ailanthone is a natural phytotoxic compound [1], [2]. The data in this article describe its pre-emergence herbicidal activity in terms of germination and growth inhibition on two model species, namely garden cress (*Lepidium sativum* L. ˈIngleseˈ) and radish (*Raphanus sativus* L. ˈTondo Rosso BIOˈ), sown on filter paper, cultivation substrate and non-sterile soil, in controlled laboratory conditions. The dose-response curve of ailanthone (Ail) on filter paper and related information are reported in Fig. 1, Fig. 2, Table 1, and Table 2. The phytotoxicity dynamics of 1.5 and 7.5 mg L^−1^ of Ail along 30 days on filter paper is reported in Fig. 3. Fig. 4 shows the response to 7.5, 30, 60, and 90 mg L^−1^ of Ail on a cultivation substrate and on non-sterile soil. Lastly, the effect of 30, 60, and 90 mg L^−1^ of Ail in cultivation substrate during time is displayed in Fig. 5. Part of the dataset was used to calculate the index of germination and the index of growth as reported in the related paper [1].

**Table 1:** Ailanthone concentration (mg L^−1^) required to reduce by 10%, 50% and 90% (ED_10_, ED_50_ and ED_90_, respectively) radish total germination and their lower and upper limits of the confidence interval.

	Radish	Lower limit	Upper limit
ED_10_	0.79	0.61	0.96
ED_50_	2.68	2.40	2.96
ED_90_	9.11	7.13	11.08

**Table 2:** Ailanthone concentration (mg L^−1^) required to reduce by 10%, 50% and 90% *(*ED_10_, ED_50_ and ED_90_, respectively) garden cress and radish root length and ED ratio with its lower and upper limits of the confidence interval.

	Garden cress	Radish	ED ratio^a^	ED ratio lower limit	Ed ratio upper limit
ED_10_	0.37	0.44	0.86ns	0.17	1.54
ED_50_	1.03	1.49	0.69*	0.51	0.88
ED_90_	2.85	5.07	0.56*	0.13	0.99

The statistical relevance is provided (ns, non-significant; *p ≤ 0.05).

a ED ratio: calculated as EDx garden cress/EDx radish.

## 2 Experimental design, materials, and methods

To assess the dose-response curve, ten seeds per each species were placed on filter paper disk (Whatman No. 1, Maidstone, UK), in a 9 cm-diameter Petri dishes. Treatments consisted in 5 mL of increasing concentrations of Ail (Baoji Herbest Bio-Tech Co., Ltd. Baoji, China) distributed as aqueous solutions (1, 1.5, 2, 2.5, 5, 7.5 and 10 mg L^−1^). Seeds treated with 5 mL of deionised water were used as control. Dishes were placed in a growth chamber at 25 °C, in dark conditions and 96 hours after the treatment, the germination (%) and the mean root length (mm) of germinated seeds per dish were recorded. Three replicates were prepared per each treatment (30 seeds).

Five seeds were placed on filter paper in 100 mL plastic flasks to assess the phytotoxicity dynamics of 1.5 and 7.5 mg L^−1^ of Ail during time. 1.7 mL of treatment or deionised water was added and flasks were placed in a growth chamber at 25 °C, with 12h of light and 12 h of dark. Ten days after treatment (DAT), the germination (%), the mean root length (mm) and the mean hypocotyl length (mm) of germinated seeds per flask were recorded. Six replicates (30 seeds) per treatment were prepared. All seedlings and non-germinated seeds were removed and new seeds were put on the filter paper. Deionised water was added to prevent dryness. The same assessment of 10 DAT was performed at 20 DAT and repeated again at 30 DAT, after replacing seedling and non-germinated seeds with new seeds.

The sensitivity of the two model species was also evaluated in non-sterile soil and in a cultivation substrate used in horticulture (Floradur® B Seed, Floragard Vertriebs-GmbH) [1]. Plastic flasks (100 mL) were filled with 20 g of substrate or soil and moistened with 5 mL of deionised water. Five seeds were then placed and treated with 1.7 mL of deionised water or Ail aqueous solutions (7.5, 30, 60, and 90 mg L^−1^). Flasks were maintained in the dark at 25 °C in a growth chamber for 96 hours; afterwards, germination (%) and the mean root length (mm) of germinated seeds per flask were recorded (Fig. 4). Three replicates (15 seeds) per treatment were prepared.

As well as was done in filter paper, the phytotoxicity dynamics of 30, 60 and 90 mg L^−1^ of Ail was also evaluated in non-sterile soil and cultivation substrate, collecting data on the germination (%), the mean root length (mm) and the mean hypocotyl length (mm) of germinated seeds per flask at 10, 20 and 30 DAT (Fig. 5). Three replicates (15 seeds) per treatment were prepared.

As for the dose-response assessment, a curve was prepared for each parameter recorded (germination and root length). In particular, concerning the germination, a separate regression analysis was performed for each species between Ail concentration (independent variable) and the total germination (proportion of germinated seeds), (dependent variable) fitting the following two parameters log-logistic model to binomial response (Equation (1)):

(1) $$\text{GT}=\;\frac{1}{1+\text{exp}\left[\text{b}\left(\text{log}\left(\text{x}\right)-\text{log}\left(\text{e}\right)\right)\right]}$$

where *GT* is the total germination expressed as a proportion of germinated seeds, *b* and *e* are the curve parameters, with *b* being the relative slope at the point of inflection *e* and *x* is the Ail concentration (in mg L^−1^). For garden cress, it was not possible to draw a regression curve as the germination did not decrease strongly at increasing rates of Ail. Regarding the root length at different Ail concentrations, data were prior transformed as a percentage of root length of the control. Afterward, for each species a separate regression analysis was performed between Ail concentration (independent variable) and the root length (dependent variable) fitting the following four parameters log-logistic model (Equation (2)):

(2) $$\text{Y}=\text{ c}+\frac{d-c}{1+\text{exp}\left[\text{b}\left(\text{log}\left(\text{x}\right)-\text{log}\left(\text{e}\right)\right)\right]}$$

where *Y* is the root length at each Ail concentrations, *b*, *c* and *e* are the curve parameters, with *b* being the relative slope at the point of inflection *e*, *c* the lower limit and *d* the upper limit, and *x* is the Ail concentration (in mg L^−1^). For both total germination and root length, the regression analysis was performed using the function *drm* of the add-on package *drc* of the *R* software [3]. The effective concentration required to reduce by 10%, 50% and 90% the total germination and the root length (ED_10_, ED_50_, ED_90_) was calculated using the function *ED* of the package *drc*. The function *EDcomp* of the package *drc* was used to calculate the ratio between the ED values of the two species (ED ratio) only for root length and to test the significance of differences of ED_10_, ED_50_, and ED_90_ between the two species. Differences were considered significant when the confidence interval of ED ratio at p ≤ 0.05 did not included one.

All the other data were analyzed through SPSS software (SPSS Inc., version 25, Chicago, Illinois). The homogeneity of variance (Levene test) was tested, then one-way ANOVA was used to analyse the phytotoxic effect of Ail on model species, separating means according to Tukey post-hoc test (*p* < 0.05).
